# Oral Immunization With Plant-Based Vaccine Induces a Protective Response Against Infectious Bursal Disease

**DOI:** 10.3389/fpls.2021.741469

**Published:** 2021-11-18

**Authors:** María Soledad Lucero, Silvina Chimeno Zoth, Juan Jaton, María José Gravisaco, Silvina Pinto, Matías Richetta, Analía Berinstein, Evangelina Gómez

**Affiliations:** ^1^Laboratorio de Inmunología y Vacunas Aviares, Instituto de Agrobiotecnología y Biología Molecular, INTA-CONICET, Buenos Aires, Argentina; ^2^Cátedra de Patología, Facultad de Ciencias Veterinarias, Universidad de Buenos Aires, Buenos Aires, Argentina; ^3^Gerencia de Gestión Estratégica de Procesos Complementarios, Centro de Investigación en Ciencias Veterinarias y Agronómicas, INTA, Buenos Aires, Argentina

**Keywords:** plant-based vaccine, infectious bursal disease, VP2, oral immunization, chickens

## Abstract

Infectious bursal disease virus (IBDV) is the etiological agent of an immunosuppressive and highly contagious disease that affects young birds causing important economic losses in the poultry industry worldwide. We have previously developed a plant-based vaccine candidate for infectious bursal disease (IBD) that is able to protect against infection with IBDV when administered through intramuscular (im) route. Given that oral vaccination is non-invasive and stimulates the immunity of the mucosal gastrointestinal surface, the initial site of contact and entry of IBDV, the aim of this work was to study if our immunogen was also able to elicit a protective immune response when orally administered. We demonstrated that 85% of the animals that received two oral doses of the vaccine formulation and all animals that were orally boosted after an im prime scheme developed virus neutralizing antibodies and were protected against IBDV infection, evidenced by the bursa/body weight (BB) ratio, absence of T-cell infiltration, and low viral load in bursa. Although mild to moderate bursal damage was observed in some of these animals, these lesions were not as severe as the ones observed in challenged control groups, which also presented signs of acute inflammation, bursal atrophy, T-cell infiltration, and absence of viral clearance. These results show that two immunizations with our recombinant immunogen are able to induce a specific and protective immune response in chicken against IBDV when orally administered in a prime/boost scheme or when the oral boost follows an im prime scheme. In conclusion, our oral plant-based vaccine candidate could represent a viable alternative to conventional vaccines and is of great interest to the poultry industry.

## Introduction

Infectious bursal disease virus (IBDV) is a non-enveloped icosahedral bisegmented double-stranded RNA virus, which is a member of the *Birnaviridae* family (Dobos et al., 1979; Müller et al., 1979). It is the etiological agent of infectious bursal disease (IBD), an acute highly contagious immunosuppressive disease that affects young birds, causing important economic losses in the poultry industry worldwide both directly, through clinical signs and mortality, and indirectly, due to incremented susceptibility to other pathogens and failure in vaccination programs (Chanie and Kegne, 2014; Rautenschlein and Alkie, 2016). The IBDV is transmitted through the fecal–oral route, initiates replication in gut-associated macrophages and lymphoid cells, and later reaches the bursa of Fabricius (BF) where it infects and destroys IgM-bearing B-lymphocytes causing severe immunosuppression of primary antibody response (Sharma et al., 2000).

Infectious bursal disease virus is highly stable and resistant to many physical and chemical agents, making it difficult to eliminate from infected poultry farms; in this sense, biosecurity measures, strict hygiene management and, more importantly, effective immunization programs, are fundamental to prevent infection in production facilities (Müller et al., 2012; Dey et al., 2019).

There have been many developments of IBDV recombinant subunit vaccines based on the expression of the capsid protein VP2, which contains the major neutralizing epitopes, in a variety of heterologous systems (Lucero et al., 2012; Müller et al., 2012; Rautenschlein and Alkie, 2016). Most of them have proven to be effective when parenterally administered, but fewer studies have shown the efficacy of VP2 oral vaccines. Oral immunization is considered as one of the most convenient routes of vaccination not only because it provides painless, easy, and safe administration but also because it stimulates the immunity of mucosal gastrointestinal surface, initial site of contact, and entry for numerous pathogens, such as IBDV. However, oral vaccination is challenging due to the fact that antigens have to endure the harsh environment of the gastrointestinal tract (GIT) in order to elicit an efficient immune response; moreover, orally-delivered subunit vaccines tend to have limited and short-lived immunogenicity. Consequently, mucosal vaccination usually requires a larger amount of antigen to compensate the degradation by gastric acid and proteases present in the GIT, multiple boosts, and/or the coadministration of adjuvants (Streatfield, 2006; Vela Ramirez et al., 2017).

Expression systems that can produce recombinant VP2 suitable for oral delivery with little or no purification, such as lactic bacteria (Liu et al., 2018; Maqsood et al., 2018; Wang et al., 2019), yeast (Arnold et al., 2012; Taghavian et al., 2013), and plants (Wu et al., 2004, 2007) have proven to be attractive platforms for the development of IBDV edible vaccines. Among them, plants offer additional advantages, such as the absence of animal pathogens in the production process, improved product quality and safety, reduction of manufacturing costs, and simplified scale-up (Rage et al., 2020).

We have previously developed a plant-based vaccine candidate for IBD by means of a transient VP2 expression in *Nicotiana benthamiana* (Gómez et al., 2013) and demonstrated that it was able to protect chicken against infection with IBDV when 7.5 μg of VP2 were administered in a prime/boost scheme through the intramuscular (im) route but not when delivered by the oral or intranasal routes (Lucero et al., 2016). Hence, the objective of the present study was to evaluate the effectiveness of a larger dose of immunogen to prime scheme or boost a protective immune response by the oral route.

## Materials and Methods

### VP2 Transient Expression and Antigen Preparation

Transient expression was performed by infiltrating 5- to 6-week-old greenhouse-grown *Nicotiana benthamiana* leaves with a suspension of recombinant *Agrobacterium tumefaciens* strain, GV3101 harboring pEAQ-VP2 vector as previously described (Lucero et al., 2019; Gómez et al., 2020). Briefly, the recombinant bacteria were cultured in Luria–Bertani medium containing 100 μg/mL Kanamycin, 100 μg/mL of Rifampicin, and 50 μg/mL of Gentamicin for 32 h at 28°C, pelleted and resuspended in an infiltration solution [10 mM of morpholinoethanesulfonic acid (MES), pH 5.5; 10 mM MgSO_4_, and 100 μM acetosyringone] to an OD_600_ of 0.8–1. Agroinfiltrated leaves were harvested 5 days post inoculation, and blended with three volumes of chilled phosphate-buffered saline (PBS) with a protease inhibitor cocktail (Roche, Mannheim, Germany). Leaf extract was filtered through gauze, centrifuged for 20 min at 10,000 × *g*, and concentrated by ultracentrifugation (Gómez et al., 2020). Then, a supernatant was filtered through a 0.45 μm membrane in a filter device and loaded above 25% (14 mL) and 70% (3.5 mL) w/v sucrose layers. After 3 h of ultracentrifugation at 41,000 rpm in a 45 Ti rotor (Beckman) at 4°C, the interface and bottom fractions were pooled and samples were kept at −80°C until use. Negative control sample was obtained from pEAQ-green fluorescent protein (GFP) agroinfiltrated leaves that underwent the same protocol as described above.

### Detection and Quantification of the Recombinant Protein

The expression of VP2 was analyzed by western blot assays. Briefly, the extracted proteins were separated in a 10% SDS-PAGE and blotted onto a nitrocellulose membrane. Recombinant protein was identified using an anti-VP2 rabbit polyclonal antibody. Quantification of VP2 in a concentrated plant extract was estimated by comparison with a standard curve of bovine serum albumin (BSA). Briefly, serial dilutions of BSA (200, 100, 50, 25, and 12.5 μg/ml) were loaded and resolved in a 10% SDS-PAGE along with the sample of interest. After Coomassie Brilliant Blue staining, bands were analyzed with Gel-Pro Analyzer software v3.1.

### Animals

Embryonated eggs laid by specific pathogen-free White Leghorn hens were purchased from the Instituto Rosenbusch S.A. (Buenos Aires, Argentina) and hatched in an automatic incubator (Yonar, Buenos Aires, Argentina). The chickens were kept in individual cages with food and water *ad libitum*. All procedures were performed in agreement with institutional guidelines and approved by the Institutional Animal Care and Use Committee (CICUAE—CNIA—INTA, Approval no. 1/2021).

### Experimental Design

Fourteen-day old chickens were randomly divided into six groups. Animals were immunized with concentrated plant extract containing recombinant VP2 in a prime/boost scheme at 0 and 14 days post immunization (dpi) through the oral or im routes as described in Table 1. Oral immunization was performed by holding the beaks of the chicken open, administering a concentrated plant extract, through an automatic 1 ml pipette, on the tongue and allowing the chicken to swallow. Intramuscular immunization was performed by injection in the leg muscle. Three weeks after boost (35 dpi) the chickens were orally challenged with 10^2^ EID_50_ of a classical virulent Argentinian field strain isolated from broiler chicken in 2012 kindly provided by Dr. Vagnozzi, Instituto de Virología, CICVyA, INTA. The GFP-immunized and non-immunized challenged animals served as infection controls, while non-immunized and non-challenged animals were the healthy control groups. One week after IBDV challenge (42 dpi), the animals were humanly euthanized and bursas were removed to determine BB ratio and to perform histopathological observation, quantification of viral load by real-time quantitative reverse transcription PCR (RT-qPCR), and T-cell infiltration by flow cytometry. The chickens were bled by the wing vein at 11, 21, and 32 dpi to assay seroconversion.

**TABLE 1:** Experimental design.

Group	Prime	Boost	Challenge
Healthy (*n* = 5)	–	–	–
VP2 oral (*n* = 7)	oral VP2 (60 μg)	oral VP2 (60 μg)	10^2^ EID_50_ IBDV
VP2 im/oral (*n* = 7)	im VP2 (30 μg)	oral VP2 (60 μg)	10^2^ EID_50_ IBDV
VP2 im (*n* = 7)	im VP2 (30 μg)	im VP2 (30 μg)	10^2^ EID_50_ IBDV
GFP (*n* = 7)	oral GFP	oral GFP	10^2^ EID_50_ IBDV
Challenged (*n* = 5)	–	–	10^2^ EID_50_ IBDV

*Fourteen-day specific pathogen free chicken were immunized in a prime/boost scheme at 0 and 14 days post immunization (dpi). Animals were challenged 3 weeks after boost and euthanized 7 days post challenge.*

### Evaluation of Humoral Response

Sera were tested for specific anti-VP2 antibodies using an indirect in-house ELISA based on IBDV SVP (Gómez et al., 2020). Briefly, 96-well MaxiSorp™ Nunc™ flat-bottom plates (Thermo Fischer Scientific, MA, United States) were coated with 95 ng of SVP per well in 0.1 M carbonate–bicarbonate buffer, with pH of 9.6, overnight at 4°C. After blocking with 4% skimmed milk in PBS-T (0.05% Tween 20), the plates were subsequently incubated with a 1:400 dilution of sample sera, washed and incubated again with a 1:4000 dilution of goat anti-chicken IgG antibodies coupled to horseradish peroxidase (Bethyl Laboratories, United States). A revealing step was performed using ABTS substrate (Sigma-Aldrich, MO, United States)-H_2_O_2_ in a citric acid buffer, having pH 5. Reading was done at 405 nm after 20 min of incubation. Samples with absorbance above the cut-off value of 0.249 were considered positive.

### Seroneutralization Assay

Seroneutralization assay was performed as previously described (Lucero et al., 2019). Briefly, the sera were inactivated for 30 min at 56°C, two-fold serially diluted in a culture medium (50% MEM-D, 50% MEM-E, HEPES 1X, pH 7.4) and incubated with 100 TCID_50_ of IBDV strain Winterfield for 1 h at 37°C in a 96-well plate. Subsequently, 100 μl of a cell suspension of 1 × 10^6^ VERO cells/ml were added to each well. Cells were cultured at 37°C, 5% CO_2_ for 4 days, when cytopathic effect was observed. Virus neutralizing antibody titers were calculated as the inverse of the last dilution showing no cytopathic effect. Two sera belonging to hyperimmunized hens were used as positive controls.

### Bursa/Body Weight Ratio

Body weight and bursa weight were used to calculate the BB ratio according to the following formula:


B⁢B⁢r⁢a⁢t⁢i⁢o=[b⁢u⁢r⁢s⁢a⁢w⁢e⁢i⁢g⁢h⁢t⁢(g)/b⁢o⁢d⁢y⁢w⁢e⁢i⁢g⁢h⁢t⁢(g)]×1000

### Histopathological Observation of Bursa

Bursal samples were placed in 10% neutral and paraffin embedded buffered formalin. Sections of the paraffin embedded BF were stained with hematoxylin and eosin following standard histological procedures. The stained sections were microscopically examined for the presence of bursal lesions by light microscopy. The severity of bursal depletion and necrosis was determined by evaluating each characteristic in 5 fields at 100X and scoring them from 1 to 5, where 1 = normal BF, 2 = <25%, 3 = 25–50%, 4 = 50–75%, and 5 = 75–100% of affected tissue. The sum of both parameters resulted in the classification of bursal lesion as normal (2), mild (3–4), moderate (5–7), and severe (8–10). Additionally, the degree of acute inflammation was determined by the assessment for the presence of edema and heterophile infiltration followed by scoring and categorization using the same criteria as described before.

### Lymphocyte Isolation and Flow Cytometry Analysis

Lymphocytes were isolated from bursal samples and were used to study T-cell infiltration by flow cytometry as previously described (Carballeda et al., 2011). Briefly, bursas were mechanically disrupted in RPMI 1640 and cellular suspensions were passed through a 40 μm mesh (Cell Strainer, BD). Mononuclear cells were isolated by centrifugation over a Histopaque density gradient. About 1 × 10^6^ cells per well were seeded on a 96-well plate and stained with different combinations of antibodies. Monoclonal antibodies (mAbs) (CD3-SPRD, CD4-PE, CD8α-FITC, Bu-PE) were purchased from SouthernBiotech (Birmingham, AL, United States). Cell suspensions were analyzed with a FACSCalibur flow cytometer (BD Biosciences, San Jose, CA, United States) and CellQuest software. The lymphocyte gate was defined by the forward/side scatter characteristics of the cells and 50,000 events were analyzed for each sample. Individual values of all experimental groups were normalized to the mean values of unchallenged healthy group.

### Viral Load Quantification in Bursa

Total RNA was extracted from pieces of bursa stored in TransZol (TransGen Biotech Co., Ltd., Beijing, China) according to the protocol provided by the supplier. The quantity and quality of the extracted RNA was determined using NanoDrop™ ND-1000 (Thermo Fisher Scientific, Wilmington, DE, United States) and agarose gel electrophoresis. The complementary DNA (cDNA) synthesis and qPCR was performed in a single step reaction utilizing Luna^®^ Universal Probe One-Step RT-qPCR Kit (New England Biolabs, MA, United States) according to the protocol of the manufacturer. Primers used for retrotranscription and amplification were VP1f: 5′CCAACACACCTCATGATCTC3′ and VP1r: 5′GTCAATTGAGTACCACGTGTT3′ that amplify a product of 222 bp belonging to IBDV *vp1* gene. Number of viral copies per microgram of RNA was calculated by extrapolation with a standard curve generated by qPCR from ten-fold dilutions of a plasmid containing the amplified *vp1* fragment ranging from 10^7^ to 10^2^ copy numbers.

### Statistical Analysis

Statistical analysis was performed using one-way ANOVA when normality and homoscedasticity were confirmed by Shapiro–Wilk and Levene tests, respectively. Comparison among means was done by Fisher LSD test. When assumptions were not fulfilled, Kruskal–Wallis non-parametric test was applied followed by Wilcoxon pairwise comparison. All the analyses were done using R 3.4.1 (R Core Team, 2013) and agricolae package (de Mendiburu and Yaseen, 2020).

## Results

### Evaluation of Antigen Expression

**FIGURE 1:**
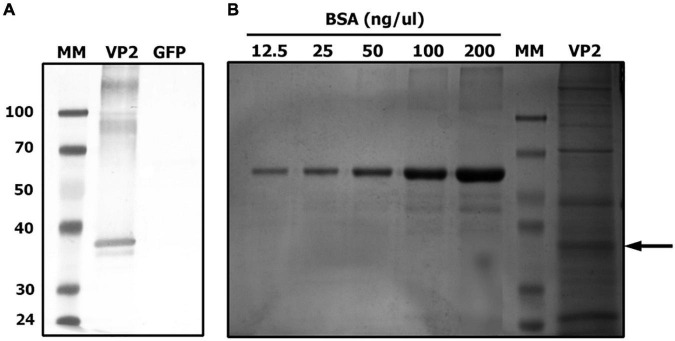
VP2 transient expression in *Nicotiana benthamiana* plants. Extracted proteins from VP2 or GFP (negative control) agroinfiltrated leaves were separated on a 10% SDS-PAGE. **(A)** Identification of recombinant VP2 by western blot using an anti-VP2 antiserum. **(B)** Quantification of VP2 by comparison with a standard curve of bovine serum albumin (BSA) after Coomassie Brilliant Blue staining. MM, molecular marker. Arrow indicates VP2 band.

Before performing chicken experiments, the expression of recombinant VP2 in a concentrated plant extract was confirmed by Western blot and quantified by SDS-PAGE followed by Coomassie Brilliant Blue staining. A specific band corresponding to the mature VP2 was observed at the expected size and the estimated concentration of VP2 antigen in the plant extract was approximately 30 μg/ml (Figure 1).

### Evaluation of Antibody Response

**FIGURE 2:**
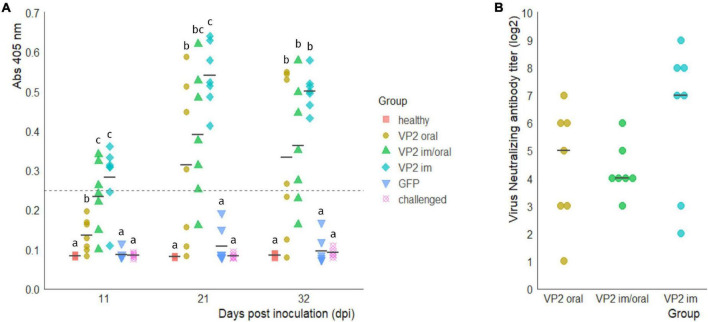
Anti-VP2 humoral response. **(A)** Specific antibody levels in the sera of immunized animals at 0 and 14 days post immunization (dpi) with VP2 through different routes or green fluorescent protein (GFP) as an unrelated antigen and of non-immunized animals (healthy and challenged groups) were measured at 11, 21, and 32 dpi with an in-house ELISA. Individual absorbances at 405 nm and mean values (black line) for each group at the different time points are shown. Levels above the cutoff point, 0.249 (dotted line) were considered positive. Different letters indicate significant differences among groups within each time point (Kruskal–Wallis test and Wilcoxon pairwise comparison, *p* < 0.05). **(B)** Individual virus neutralizing antibody titers (log_2_) in VP2 immunized animals at 32 dpi, calculated as the inverse of the last dilution showing no cytopathic effect, and median for each group (black line). No significant differences were observed between groups (Kruskal–Wallis test *p* > 0.05). Log_2_ VN titers from GFP, healthy and control groups were ≤1 and are not shown.

To assess the ability of the recombinant immunogen to elicit a humoral response when orally administered, the animals were immunized either in an oral prime/boost scheme with 2 ml of concentrated plant extract containing 60 μg of VP2 (VP2 oral group) or primed im with 30 μg of VP2 and orally boosted (VP2 im/oral group). Chicken that received two im injections of the immunogen (VP2 im group) acted as positive control since we have already demonstrated that this scheme is able to elicit a humoral and protective immune response (Lucero et al., 2016). On the other hand, the birds in GFP group were orally administered a plant extract containing GFP as a non-related antigen while healthy and challenged groups, did not receive any immunization. Sera were analyzed for the presence of specific antibodies against VP2 using an in-house ELISA developed and validated in our laboratory. Figure 2A shows that as early as 11 dpi, after one im immunization, seven chickens, three in VP2 im/oral and four in VP2 im groups, were positive for the presence of specific antibodies (Abs at 405 nm > 0.249) and were significantly different from the rest of the groups (*p* < 0.005 vs. healthy, GFP and challenge and *p* < 0.01 vs. oral). At this time point, despite significant differences with healthy, GFP, and challenged groups (*p* < 0.05), all animals in VP2 oral group were considered negative for VP2 antibodies since absorbances were below the cut-off value. However, at 21 dpi, after the second oral dose of VP2, the antibodies were detected in four out of the seven animals in this group and their levels remained high until challenge. Furthermore, VP2 antibody titers increased after boost in VP2 im/oral and VP2 im groups and three additional animals in each group were seroconverted. Although VP2 antibody titers were higher and more homogenous in VP2 im group at every time point, no significant differences were observed with VP2 im/oral during the course of the experiment or with VP2 oral group at 32 dpi. As expected, GFP-immunized and non-immunized animals in healthy and challenged groups had undetectable levels of specific VP2 antibodies and were significantly different from VP2 immunized groups throughout the trial (*p* < 0.05). Additionally, virus neutralizing (VN) antibody titers were measured at 32 dpi. Figure 2B shows that VP2-immunized animals had serum antibodies that are capable of neutralizing IBDV infection in VERO cell culture compared to GFP-immunized or non-immunized animals (log_2_ VN titers ≤1, data not shown). Among VP2 groups, although not significantly different, VP2 im animals displayed the highest VN titers. However, there were not as high as VN titers in the two positive control sera belonging to hyperimmunized hens (log_2_ VN titers 11 and ≥13, data not shown).

### Post Challenge Analysis and Evaluation of Bursal Lesions

**FIGURE 3:**
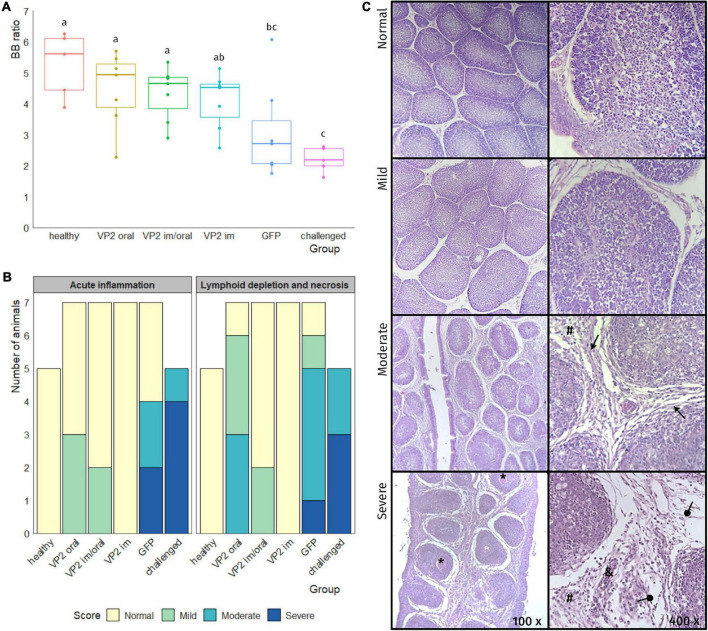
Evaluation of bursa after challenge. One week after the challenge, the animals were euthanized and bursas were removed, weighted, and paraffin embedded and stained with hematoxylin/eosin. **(A)** Individual bursa/body weight (BB) ratios determined by the formula (*bursaweight*(*g*)/*bodyweight*(*g*))× 1000 (dots) as well as box plots representing data distribution are shown for each group. Different letters indicate significant differences among groups (one-way ANOVA test and Fisher LSD *post hoc* test, *p* < 0.05). **(B)** Number of animals in each group was classified into bursal lesion categories: Normal, mild, moderate, and severe according to the degree of acute inflammation and the degree of lymphoid depletion and necrosis. **(C)** Representative photos of bursal lesions observed within each category, where variable degree of lymphoid depletion together with edema (round arrow), fibrosis (triangular arrow) heterophile infiltration (#), necrosis (*), and vasculitis (&) can be seen.

Three weeks after boost, all the animals, except the healthy control group, were challenged with a classical virulent IBDV Argentinian field strain isolate (10^2^ EID_50_) and sacrificed 7 days later. With the exception of one bird in VP2 oral group, no morphological changes or significant macroscopic lesions were observed in the bursa of animals vaccinated with VP2, independent of the route. On the other hand, bursas from GFP or challenged groups displayed typical signs of IBDV infection including atrophy, yellowish appearance, and gelatinous exudate on the serosa (data not shown). Although the BB ratios were heterogeneous within VP2 vaccinated groups, no significant differences were observed between these and healthy control groups (Figure 3A). On the contrary, they were significantly different in BB ratios from GFP [*p* < 0.05 except for VP2 im vs. GFP which was non-significant (ns)] or unimmunized challenged (*p* < 0.01) animals that, with the exception of one GFP chicken, presented smaller bursas. Regarding the histological observation of bursal lesions, all VP2 im and five VP2 im/oral chicken displayed normal bursas undistinguishable from healthy unchallenged animals, while the remaining two chickens in VP2 im/oral group had mild bursal lesions due to the presence of minor lymphoid depletion (Figures 3B,C). In contrast, the majority of VP2 oral group exhibited some degree of bursal lesion (three mild and three moderate) which was characterized by variable extent of lymphoid depletion but the absence of necrosis. Still, these animals were healthier than challenged birds which showed moderate to severe bursal damage with both lymphoid depletion and necrosis. Moreover, these animals presented extensive edema, high heterophile infiltration, and vasculitis, all characteristics of acute inflammation. Although GFP group did not show bursal lesions as severe as the challenged group, there were still more pronounced than VP2 oral group and, unlike this last group, the GFP animals also presented signs of moderate to severe acute inflammation (Figures 3B,C).

### Evaluation of T-Cell Infiltration in Bursa After Challenge

**FIGURE 4:**
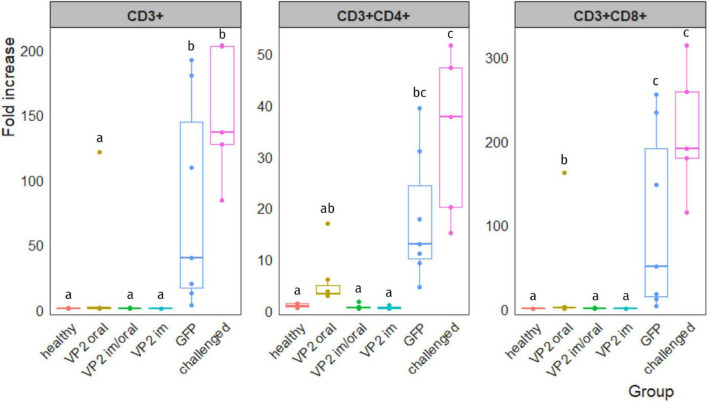
T-cell infiltration in IBDV-infected bursa. Leukocytes were isolated from bursa, stained with different combinations of antibodies and analyzed by flow cytometry. Lymphocyte population was gated according to their size and complexity. Results are expressed as the fold increase of each sample normalized with the mean value of the corresponding T-cell subpopulation obtained from healthy unchallenged chicken. Individual fold-increase values (dots) as well as box plots representing data distribution are shown for each group. Different letters indicate significant differences among groups (Kruskal–Wallis test and Wilcoxon pairwise comparison, *p* < 0.05).

After infection, IBDV replication in the bursa involves an infiltration of T lymphocytes into this organ, particularly CD8^+^ cytotoxic T cells (Tanimura and Sharma, 1997). Hence, the level of T-cell infiltration in the bursa after IBDV challenge could provide an indication of vaccine protective efficacy. Results are shown in Figure 4 and they are expressed as the fold increase of each individual sample normalized with the mean value of unchallenged healthy chicken. All animals vaccinated with the recombinant immunogen, regardless of the vaccination route, displayed low levels of T-cell infiltration with the exception of one bird from VP2 oral group which showed a fold increase of infiltrating CD3^+^, CD3^+^CD4^+^, and CD3^+^CD8^+^ cells of 121.0, 17.0, and 161.8, respectively. In spite of this, VP2 oral group was not significantly different from healthy, VP2 im/oral, and VP2 im groups when comparing total CD3^+^ and CD3^+^CD4^+^ T lymphocytes. As expected, the challenged group had high and significant levels of T-cell infiltration with fold increase medians (interquartile range) of 136.5 (127.3–202.7) (CD3^+^), 37.7 (20.1–47.2) (CD3^+^CD4^+^), and 190.7 (179.9–258.3) (CD3^+^CD8^+^). The GFP group on the other hand, was highly heterogeneous; still, the T-cell infiltration was significantly different from healthy (*p* < 0.005), VP2 im/oral (*p* < 0.005), and VP2 im (*p* < 0.001) groups, and in the case of CD3^+^ and CD3^+^CD8^+^, from VP2 oral group as well (*p* < 0.05), while no differences were observed in the challenged animals.

### Viral Load in Bursa

**FIGURE 5:**
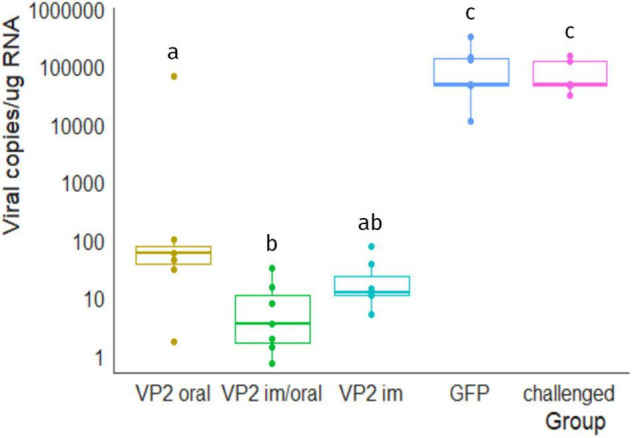
Viral load in bursa 7 days post challenge. Number of viral copies/μg of bursal RNA were estimated by RT-qPCR. Individual values (dots) as well as box plots representing data distribution are shown for each group. Different letters indicate significant differences among groups (Kruskal–Wallis test and Wilcoxon pairwise comparison, *p* < 0.05). No viral genome was detected in unchallenged healthy animals (data not shown).

Finally, viral load in bursa was quantified by RT-qPCR in order to determine if IBDV was able to reach and/or replicate in its target organ. Figure 5 shows the number of IBDV copies per microgram of bursal RNA in each animal, calculated by extrapolation with *vp1* fragment standard curve. Unsurprisingly, all animals in the GFP and challenged groups had a high number of viral copies in bursa, ranging from 1.1 × 10^4^ to 3.3 × 10^5^ and from 3 × 10^4^ to 1.5 × 10^5^, respectively, which were significantly different from VP2-vaccinated animals (GFP vs. VP2 oral, VP2 im/oral and VP2 im *p* < 0.01, challenged vs. VP2 oral, VP2 im/oral and VP2 im *p* < 0.05). Despite one chicken that exhibited more than 66,000 copies of IBDV/μg RNA, VP2 oral animals contained less than 100 viral copies/μg RNA. VP2 im/oral and VP2 im animals also showed low numbers of viral copies, with median (IQR) values of 3.5 (1.6–11.5) and 11.9 (10.9–26.6) viral copies/μg RNA, respectively. As expected, virus was not found in the bursa of unchallenged healthy animals (data not shown).

## Discussion

The plant-made vaccine field started more than three decades ago with the promise of “cheap, edible vaccines,” however, this goal has not yet been achieved (Rybicki, 2010; Chan and Daniell, 2015). While the idea of using edible plants or fruits to deliver vaccines is still very appealing, it raises some issues regarding quality control and dosage. Therefore, although oral dosing is still a desirable feature, the product itself might need to be processed to some extent, formulated, and given supervision so as to ensure reproducible effects (Rybicki, 2010). In spite of this, plant technology has proven its worth as an affordable, easily scalable production platform for vaccines, and it is particularly attractive for industries with low profit margins and less stringent regulatory hurdles, such as the veterinary industry (Chan and Daniell, 2015; Rybicki, 2018).

We have previously demonstrated that VP2 transient expression in *N benthamiana* represents a viable platform for the production of a safe, economic, and efficacious vaccine against IBD. Our immunogen, consisting of plant protein extract, can both achieve protection against disease when administered im to young chicken and induce uniform long-lasting high titer antibody response in breeder hens in order to provide effective passive immunity to the offspring (Lucero et al., 2016, 2019; Richetta et al., 2017). In the present work, we continued to study the efficacy of the immunogen and demonstrated that it can also elicit a protective immune response when orally administered. This suggests that, although the antigen is not bioencapsulated by plant cell wall, since our formulation consists of extracted proteins, VP2 is able to resist degradation in the digestive tract of chicken until it is taken by M cells which allows it to reach immunocompetent cells in the gut-associated lymphoid tissue (Taghavian et al., 2013). This resistance in the GIT might be explained by the fact that, as recently demonstrated by us and other authors, VP2 is able to correctly self-assemble into IBD-SVP in plant cells (Gómez et al., 2020; Marusic et al., 2021) that are very stable under harsh conditions (Taghavian et al., 2012; Gómez et al., 2020).

Here we showed that two oral immunizations with the recombinant immunogen containing 60 μg of VP2 induced a systemic humoral response in four out of seven animals with VN antibody titers between 32 and 128 while the other three animals had VN titers ≤8. Given that other studies showed that four oral immunizations with 500 μg of purified IBDV-SVP plus adjuvant were only able to elicit positive IgY response in two out of five animals (Taghavian et al., 2013), our immunogen seems very promising; moreover, we have not discarded the fact that a third dose or higher immunizing doses, could be able to seroconvert all animals. Despite the fact that not all chicken had specific anti-VP2 antibodies and that VN titers might not be as high as the ones observed when the immunogen is im administered, we were able to see protection against viral infection evidenced by BB ratio, T-cell infiltration, and viral load in bursa, in all but one animal. Although neutralizing antibodies have been considered as the most relevant tool to protect against IBDV infection, there is evidence that protection can be achieved in their absence, suggesting that other mechanisms might also be relevant in the defense against IBDV (Yeh et al., 2002; Ingrao et al., 2013; Zanetti et al., 2016). On the other hand, in this experiment, we did not measure mucosal IgA in the intestine, which is one of the desirable immune responses pursued by oral vaccines. It is likely that oral immunization might have elicited a mucosal IgA response reducing primary viremia gut-associated lymphoid tissues (Rautenschlein and Alkie, 2016) and we intend to evaluate this in future experiments. For most of the parameters evaluated, no significant differences were observed between the three immunizations schemes, although prime/boost schemes that included im immunizations tended to perform better, particularly in inducing systemic humoral response. When differences were observed between VP2 groups, they were mostly due to the one infected chicken in VP2 oral group which was an outlier. It is possible that this animal might have not achieved a proper uptake of the antigen dose that is required to prompt an immune response. This reflects the difficulty of ensuring consistent dosage to get homogeneous results with oral vaccination.

We did observe, however, that VP2 immunization scheme had an effect on the degree of bursal lesions caused by IBDV challenge. While VP2 im animals were undistinguishable from healthy unchallenged ones, two VP2 im/oral animals showed mild lymphoid depletion and inflammation, and more notably, a larger proportion of VP2 oral chicken displayed mild to moderate bursal damage. Still, the extent of bursal damage and degree of inflammation were not as serious as the ones observed in GFP and challenged groups. It was not striking that im administered VP2, even in lower doses, was more effective than the orally administered antigen in preventing bursal lesions, since it has been already observed in other reports (Taghavian et al., 2013); however, it was surprising that this difference between VP2 groups was not reaffirmed by the rest of the parameters assayed.

As previously mentioned, we had already attempted mucosal vaccination against IBDV with this immunogen but were unsuccessful. We hypothesized that the dose of VP2, although enough to achieve protection when given im, was too low for oral vaccination (Lucero et al., 2016). Increasing eight times the antigen amount (60 μg of VP2) accomplished the desired result with only two immunizations. Other studies have also shown that large amounts of antigen or multiple doses are generally required to elicit efficient protection through the oral route. For instance, five oral doses at 3-day intervals of soluble VP2 expressed in *Arabidopsis thaliana* (11.44 μg of VP2 in total) were needed to induce an antibody response and 80% of protection against challenge (Wu et al., 2004), while four doses of 5 g of transgenic rice seeds expressing VP2 (between 5 and 10 mg) induced neutralizing antibodies against IBDV and protected 83.33% of immunized animals against challenge (Wu et al., 2007). Moreover, four doses of orally administered *Pichia pastoris* producing VP2, containing 400 μg or 4 mg of viral protein, or oral delivery of 500 μg purified yeast-derived antigen induced a protective immune response against IBDV in chicken which increased their survival rates from 60 to 100% compared to 40% in the control groups. Despite the survival rate, some degree of histopathological bursal lesions and viral antigen was found in the bursa of challenged animals (Taghavian et al., 2013). In other reports, oral administration of 1–3 mg of VP2 contained in dried and heat-killed *Kluyveromyces lactis* mixed with chicken feed was able to protect only 10% of the animals from B-lymphocyte depletion in bursal follicles after challenge even though the animals were pretreated with saponin, an oral adjuvant, before immunization (Arnold et al., 2012). Some of the best outcomes of oral vaccination against IBDV were obtained when using bacterial systems displaying VP2. Three immunizations with 10^9^ colony-forming units of *Lactobacillus plantarum* expressing VP2, each of them achieved by oral gavage to chicken for three consecutive days, was able to attain 100% survival and 87.5% protection rates against challenge (Maqsood et al., 2018). Moreover, one-time vaccination with *Lactococcus lactis* expressing a fusion protein constructed from the RCK protein of *Salmonella enterica* and VP2 induced the production of a specific immune response characterized by neutralizing antibodies that provided full protection against vvIBDV (Wang et al., 2019). Lastly, one oral administration of *L. lactis* co-expressing the outer membrane protein (Omp) H of the microfold (M) cell-targeting ligand and VP2 protected 80% of the animals against challenge. These successful results might be owing to the fact that these vaccine candidates contain bacterial PAMPS that can adjuvate the immune response against VP2. Our immunogen did not require formulation with additional adjuvants, which could be due to the presence of *N. benthamiana* foliar extract compounds which have been shown to have immunomodulatory properties and adjuvant-like effects (Di Bonito et al., 2009) and/or to traces of *Agrobacterium*. Still, we do not discard that the addition of appropriate mucosal adjuvants or carriers that bind receptors in the GIT to deliver target molecules more precisely could enhance the immune response elicited by our oral vaccine candidate while reducing the volume of concentrated plant extract that would be necessary to achieve a protective immunity against IBDV. In this regard, increasing VP2 expression in *N. benthamiana* and thus in the final formulation, is also a desirable goal.

Overall, these results show that two immunizations with our recombinant immunogen are able to elicit a protective immune response in chicken against IBDV when orally administered in a prime/boost scheme or when the oral boost follows an im prime scheme. Given that the most expensive part of the production of plant-produced proteins is the downstream processing (Wilken and Nikolov, 2012), our oral plant-based vaccine candidate which requires minimal processing could be of great interest for the poultry industry where there is considerable pressure to keep costs low, but regulatory burden for vaccine approval is lower than for human products. Still, there is a long way to go in order to establish how this recombinant vaccine could be applied in large-scale immunization since individual oral administration does not seem to be practical for intensive farming. Given that IBDV VP2 is a very stable protein, we do not discard the possibility of mixing our immunogen with the drinking water as it is usually done with conventional live vaccines. Spray vaccination in which the immunogen attaches to mucosa cells of the eyes and upper respiratory tract of the chicken or is ingested during preening process could be another interesting means of administration. In both the cases, large amounts of protein would be required in order to ensure appropriate dosage for all chicken or otherwise, consistent vaccination would not be achievable since it is impossible to control the amount of immunogen that is uptaken.

## Conclusion

Although more work needs to be done in order to find a suitable way of administration of the recombinant immunogen in large-scale immunization programs, we believe that our oral plant-based vaccine candidate could represent a viable alternative to conventional vaccines for the poultry industry.

## Data Availability Statement

The original contributions presented in the study are included in the article/supplementary material, further inquiries can be directed to the corresponding authors.

## Ethics Statement

The animal study was reviewed and approved by the Comisión Institucional de Cuidado y Uso de Animales de Experimentación (CICUAE—CNIA—INTA).

## Author Contributions

ML, AB, and EG conceived and designed research. ML, EG, SC, JJ, and MR conducted the experiments. SP analyzed the histological data. MG conducted flow cytometry and analyzed the results. ML wrote the first draft of the manuscript. All authors revised and approved the manuscript.

## Conflict of Interest

The authors declare that the research was conducted in the absence of any commercial or financial relationships that could be construed as a potential conflict of interest.

## Publisher’s Note

All claims expressed in this article are solely those of the authors and do not necessarily represent those of their affiliated organizations, or those of the publisher, the editors and the reviewers. Any product that may be evaluated in this article, or claim that may be made by its manufacturer, is not guaranteed or endorsed by the publisher.
